# Tetanus

**Published:** 1887-08

**Authors:** 

**Affiliations:** Eisleben


### TETANUS.

DR. LORBUCHER, EISLEBEN.

(From *Die Allg. Hom. Zeitung.*)

TRANSLATED BY PROFESSOR A. MCNEIL, M. D., SAN FRANCISCO, CAL.

L. St——, twenty-seven years old, a combmaker. Thin and pale, but yet strong, so that he has borne the occasional hard work and the many kinds of hardships connected with his business, which his trade involves, until now without any bad effects. After working several hours before the fire, he stood in a draft.

After this for several days he felt a light drawing and stiffness in his arms and legs, and on the 10th of February tetanus and trismus set in. The efforts of the allopathic physician who was called in, continued for several days, were unavailing. Of course, Opium played the chief role.

I was called on the 14th of February and found the following picture of the disease: Looking paler than usual even, and dripping from sweat, he stood supported by two men between two chairs, the backs of which he held. Arms and legs were completely stiff, the head drawn backward, the jaws immovable, and standing a finger's breadth apart. After the painful undressing was accomplished I found the muscles on the shoulders and the gluteii were drawn into knots as large as one's fist. The abdominal muscles were contracted to the hardness of boards, and the lumbar portion of the spinal column was deeply drawn in, the muscles of the neck stiff, hard, and somewhat painful to contact. The slightest attempt to move immediately caused violent, extremely painful, jerks through the entire body ; loud talking by others and loud noises had the same effect. These jerks occurred more frequently while lying than when standing or sitting on the edge of a chair. They were so painful that he must cry out, and he complained particularly of an extremely violent pain flying from the lumbar vertebræ into the legs and down into the toes. There could be no sleep under these conditions. Pulse, respiration, and temperature were normal. Some appetite, and as the jaws were not approximated it could be gratified by fluids. However, he must swallow only small quantities at a time, otherwise the above-mentioned jerks occurred readily. Stool retarded, urine normal. The senses were unclouded, head only slightly dull.

The combination of clonic and tonic spasms caused me to pronounce the diagnosis of this unfavorable disease still more serious. The only favorable indication was the open jaws, by which we could avert starvation.

After careful consideration of all the points of the case and comparison of the symptoms, I selected Nux vom. and Stramon. to conquer the disease, the former corresponding more to the tonic spasms, the latter to the clonic ones and the concomitants. I first administered Nux vom.$^{5}$ every two hours, three or four drops. As after two days of this not the least improvement was visible, and, on the contrary, an increase of the clonic spasms was unmistakable, I gave Stramon.$^{5}$ every two hours, two drops. When two days of this treatment also availed not, and after a recomparison of the symptoms with the *Materia Medica* always confirmed my selection of the remedies, I concluded that I had not given the right potency. I resolved to test the high potency, and gave Nux vom.$^{cc}$ (Jenichen), a dose every four hours. I did not need to wait long for the result. On the third day there was a considerable decrease of the rigidity, first in the neck and then in the lower part of the spinal column, and at last in the jaw. After I had thus used Nux vom.$^{cc}$ six or eight days, a decided improvement of the tonic symptoms was unmistakable, but the clonic remained in the same intensity and disturbed the patient, particularly during sleep. So I tried Stramonium, but, made wiser by experience, I gave the thirtieth, not having at my command then any higher. And again I experienced a favorable effect. After thirty-six hours the violent jerks were less painful, and he could lie down, and had, after three weeks' deprivation, enjoyed for the first time the beneficial effects of several hours' sleep. By giving alternately (as indicated, first one, then the other) Nux v.$^{cc}$ and Stramon.$^{30}$ at longer intervals all the spasmodic phenomena gradually disappeared, except a violent starting, which always occurred when any one gave the patient his hand or touched him unexpectedly, or if an unusual sound affected his auditory nerves. On studying the *Materia Medica* I found, besides many other symptoms relating decidedly to tetanus and trismus, these also under Angusturia spuria, and immediately gave it in the twelfth, three drops, three times a day. The result was not long in coming. After using it six or eight days the last remnant of the disease had gone.

The entire cure took from February 14th to March 20th. In April he began to work, and still rejoices in good health, so that he can bear all the exertions and hardships of his occupation without any bad effects. The allopathic colleague, notwithstanding my repeated invitations, did not come to see the patient, although it offered the best opportunity for him to observe the efficacy of the infinitesimal doses.

